# Identification and Expression Analysis of the Cyclin-Dependent Kinase Inhibitor *ICK*/*KRP* Gene Family in Pepper

**DOI:** 10.3390/genes17070733

**Published:** 2026-06-25

**Authors:** Tiantian Li, Qingzhi Cui, Zhuoxuan Wu, Shan Liu, Yanlong Li, Zhuqing Zhang, Wenchao Chen, Sha Yang

**Affiliations:** 1 College of Horticulture, Hunan Agricultural University, Changsha 410128, China; litiantian011@126.com (T.L.); cuiqz@stu.hunau.edu.cn (Q.C.); wuzx0820@163.com (Z.W.); 17680379704@163.com (S.L.); yanlongli@hunau.edu.cn (Y.L.); 2 Vegetable Research Institute, Hunan Academy of Agricultural Sciences, Changsha 410125, China; cszzq@126.com (Z.Z.); wencc1974@126.com (W.C.)

**Keywords:** pepper, *ICK* gene, gene family

## Abstract

Background: Cell division plays a crucial role in plant growth and development. Cyclin-dependent kinase inhibitors (*ICK*/*KRP*) negatively regulate the cell cycle, thereby affecting cell elongation and organ development. This study aimed to systematically identify and characterize the *ICK*/*KRP* gene family in pepper, and to explore their roles in growth, development, and stress responses. Methods: Bioinformatics approaches were used for genome-wide identification, chromosomal localization, collinearity analysis, sequence characterization, promoter element prediction, and tissue-specific expression profiling of pepper *ICK* genes. Phylogenetic analysis was performed with homologs from *Arabidopsis*, *tomato*, *maize*, and *rice*. Quantitative real-time PCR and virus-induced gene silencing (VIGS) were applied to validate gene expression patterns and gene function, respectively. Subcellular localization assays were also conducted. Results: A total of six *ICK* genes were identified in pepper. They were classified into three subfamilies and distributed on different chromosomes, with one pair showing evidence of duplication. All *ICK*/*KRP*s contain the conserved Motif 1 (amino acid sequence: KIPTTREIEEFFATAEKQQQRRFIEKYNFDPVNEKPL) and were predicted to localize to the nucleus. Promoter analysis revealed cis-acting elements associated with plant development, stress responses, and hormone signaling. Expression pattern analysis indicated tissue-specific divergence and significant induction/repression under temperature stress. qRT-PCR results were consistent with transcriptome data, and expression differences were observed in materials with different stigma lengths. Subcellular localization confirmed that *Caz03g38750.1* and *Caz12g03790.1* proteins localize to both the nucleus and plasma membrane. Silencing of *CazICK1* significantly repressed stigma elongation and altered stigma morphogenesis. Conclusions: The six pepper *ICK*/*KRP* genes display distinct diversity in distribution, structure and expression, and function in plant growth, development and stress adaptation. This work not only lays a solid basis for exploring the cell cycle regulatory network of pepper and contributes to relevant theoretical research, but it also identifies key gene resources for improving stigma traits. It has great potential for application in molecular breeding to promote high yield and efficient hybrid seed production in pepper.

## 1. Introduction

Plant meristems serve as the fundamental source for shaping the spatial architecture of plants throughout the entire process of plant morphogenesis. Organ development is directly dependent on the frequency of cell division, the regulation of the cell cycle, and the quantity and size of cells. To control the cell cycle and cell division, plants must regulate the activity of Cyclin-Dependent Kinases (CDKs) during development; this is coordinated with nutritional status, hormonal signals and environmental cues. Cyclin-Dependent Kinase Inhibitors (CKIs) are a protein family that inhibits CDK activity; they modulate the progression of the cell cycle and the rhythm of organ development by binding to CDKs or CDK–Cyclin complexes.

In mammals, CKIs are divided into two families: the KIP/CIP family and the INK4 family. The KIP/CIP family includes p27Kip1, p21Cip1 and p57Kip2, which contain a C-terminal CDK inhibitory domain and can reduce the activity of cyclinD-CDK4/6 and cyclinD-CDK2 complexes. By contrast, the INK4 family specifically inhibits the cyclinD-CDK4/6 complex, thereby affecting the cell cycle and leading to diseases such as cancer. For instance, knockout of the p27Kip1 gene in this family results in infertility, deafness and other disorders in mice. In plants, two CKI families have been identified so far. The first family is homologous to the mammalian KIP/CIP inhibitor p27Kip1, which is named *ICK* (also referred to as *KRP*s, Kip-Related Proteins) in *Arabidopsis*. Revised nomenclature designates them as the *ICK*/*KRP* family. Except for the conserved C-terminal domain, *ICK*/*KRP*s are substantially different from mammalian and yeast CKIs [1]. The second family is formed by the combination of SIAMESE (SIM) from *Arabidopsis* and EL2 from *rice*, which is collectively termed the SIM/EL2 family. SIM/EL2 proteins are small peptides (approximately 14 kDa in molecular weight), and their C-terminal regions share the conserved EIEDFF sequence with *ICK*/*KRP* proteins.

Biological functions of *ICK*/*KRP*, including the overexpression of *ICK*/*KRP* genes, lead to various developmental abnormalities in plants, such as aberrant plant architecture, serrated leaves, polyembryony, and cell enlargement. Cao et al. (2018) [2] generated mutants of seven *ICK*/*KRP* family genes and observed that the ovules of these mutants produced an excessive number of megaspore mother cells, resulting in polyembryony and twin seedlings. The overexpression of the *ICK4* gene was able to complement the phenotypes of the seven mutants, indicating that these seven genes exhibit functional redundancy, which ensures the formation of a single embryo and normal plant reproduction [2]. Barrôco et al. (2006) [3] overexpressed the KRP1 gene in *rice*. Phenotypic analysis revealed that the leaves of the overexpressing plants exhibited slight shortening and reduced leaf area. Further observations showed that the number of cells on the leaf surface decreased significantly, while the cell volume increased accordingly. This study also found that overexpression of KRP1 significantly affected *rice* grain filling, leading to a reduction in the number of plump seeds and a decline in yield. Meanwhile, it was confirmed that this gene plays a key role in the transition from mitosis to the endocycle in *rice* [3]. Ajadi et al. (2019) [4]. found that KRP1 and KRP2 are mainly expressed in developing *rice* seeds, and their expression is significantly induced by exogenous abscisic acid (ABA) and brassinosteroids. The constructed KRP1 overexpression line (OxKRP1), KRP2 single knockout mutant (crkrp2), and KRP1/KRP2 double knockout mutant (crkrp1/krp2) all exhibited phenotypes of reduced seed length and width, as well as decreased grain weight. In addition, the seed germination rate and final germination percentage of the three types of plants were reduced, and the early seedling growth process was significantly delayed. The study further pointed out that such phenotypic abnormalities arise from the inhibition of cell proliferation and expansion during grain filling and germination after the homeostasis of KRP1 or KRP2 is disrupted. Furthermore, it was verified that KRP1 can interact with two cyclin-dependent kinases, CDKC2 and CDKF3, suggesting that it regulates the growth and development of *rice* seeds and seedlings by modulating the mitotic cell cycle [4]. Ruan et al. (2020) [5] cloned the gene TGW2, which corresponds to the quantitative trait locus qTGW2 controlling *rice* grain width and weight. Through yeast two-hybrid and bimolecular fluorescence complementation assays, it was confirmed that the TGW2 protein can interact with the cell cycle regulator KRP1. Moreover, TGW2 negatively regulates *rice* grain width and weight by affecting cell proliferation and expansion in *rice* glumes [5]. *ICK*/*KRP* genes are also involved in plant stress responses. As a plant cyclin-dependent kinase inhibitor, the expression of *ICK1* is significantly induced by abscisic acid (ABA). After ABA induces *ICK1* expression, the activity of Cdc2-like histone H1 kinase in *Arabidopsis* decreases, which reveals the molecular mechanism by which ABA inhibits plant cell division through regulating *ICK1* [6]. Low-temperature stress significantly upregulates the expression level of tomato *SlKRP2*, while drought stress significantly upregulates the expression of tomato *SlKRP3*; the silencing of *SlKRP5* increases the sensitivity of tomato to drought stress [7]. In soybean, polyethylene glycol (PEG) stress upregulates the expression levels of *GmKRP1a*, *GmKRP2a*, and *GmKRP4* in roots. After salt stress treatment, the expression of multiple *GmKRP* genes, including *GmKRP1a*, *GmKRP2a*, *GmKRP2b*, *GmKRP4*, and *GmKRP5*, is upregulated in both leaves and roots [8]. In eggplant, with the exception of *SmKRP3*, salt and drought stresses reduce the expression levels of other KRP family genes. Salt stress significantly induces the expression of *SmKRP3*. Subcellular localization assays confirmed that all eggplant KRPs are localized in the nucleus. Meanwhile, virus-induced gene silencing (VIGS) experiments showed that silencing of *SmKRP3* significantly increases the sensitivity of eggplant to salt stress, and the transcription levels of salt stress defense-related genes and the activities of stress-resistant enzymes decrease [9].

At present, research on *ICK*/*KRP* remains limited in the Solanaceae family. In particular, studies on the *ICK*/*KRP* genes for pepper—a vital vegetable crop in China—are almost completely lacking. Therefore, in this study, bioinformatics approaches were employed to perform genome-wide identification of *ICK*/*KRP* gene family members in pepper. We further analyzed the physicochemical properties of their encoded proteins, gene structures, and evolutionary relationships, as well as gene expression patterns during plant development and under stress treatments. This work is expected to lay a solid foundation for subsequent investigations into the biological functions of *ICK*/*KRP* genes.

## 2. Results

### 2.1. Identification and Physicochemical Property Analysis of the ICK/KRP Gene Family in Pepper

A total of six *ICK*/*KRP* genes were identified from the genome of pepper ‘zhangshugang’ using bioinformatics methods: *Caz02g27290.1*, *Caz03g38750.1*, *Caz04g19670.1*, *Caz08g21920.1*, *Caz09g12900.1* and *Caz12g03790.1*. The lengths of their protein sequences ranged from 196 to 232 amino acids. The molecular weight, grand average of hydropathicity (GRAVY), isoelectric point (pI), instability index and aliphatic index of these proteins were 21,761.67–26256.5, −1.03–0.605, 4.32–9.05, 40.02–72.06 and 52.73–69.4, respectively (Table 1). Subcellular localization prediction revealed that all these *ICK*/*KRP*s were localized in the nucleus, which was consistent with the subcellular localization of five genes in the *arabidopsis KRP* gene family (Table 2).

### 2.2. Phylogenetic Analysis of ICK/KRP Genes in Pepper, Tomato, Rice, Maize and Arabidopsis

**Figure 1:**
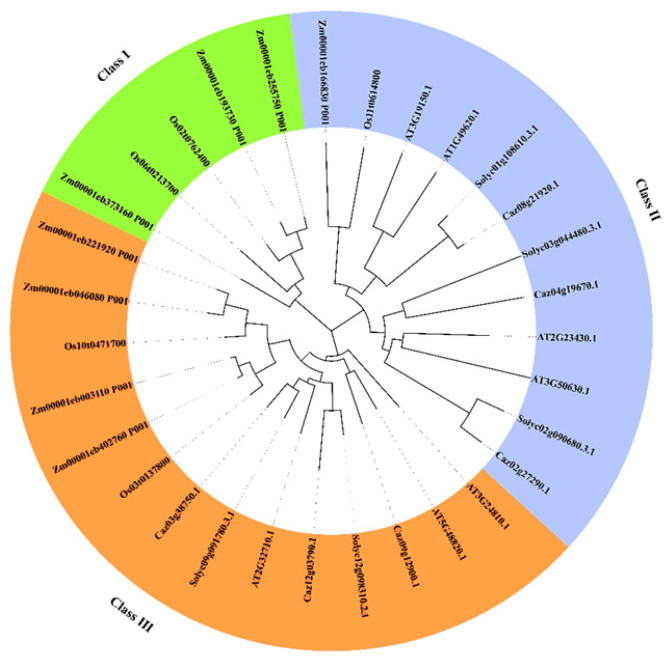
The phylogenetic tree of the *ICK*/*KRP* gene family in pepper, *arabidopsis*, *tomato*, *maize*, and *rice*.

To analyze the phylogenetic relationships between pepper *KRPs* and their homologous genes from other plant species, we constructed a phylogenetic tree of KRPs using MEGA 7.0 software with the *KRP* gene family members of *arabidopsis*, *tomato*, *maize* and *rice*. We found that the *KRP* genes from these five plant species were divided into three subclasses, namely subclass I, subclass II and subclass III (Figure 1). Subclass I was predominantly composed of the *KRP* genes from *rice* and *maize*, which indicated that there was a differentiation of the *KRP* gene family between monocotyledons and dicotyledons. In subclass II, *Caz08g21920.1* and *Caz02g27290.1* were clustered into the same clade, suggesting that *Caz08g21920.1* may share similar sequences and functions with *Caz02g27290.1*. In subclass III, four *KRP* genes (*Caz03g38750.1*, *Caz04g19670.1*, *Caz09g12900.1* and *Caz12g03790.1*) exhibited highly similar sequences.

### 2.3. Chromosomal Localization and Collinearity Analysis of ICK/KRP Family Members

**Figure 2:**
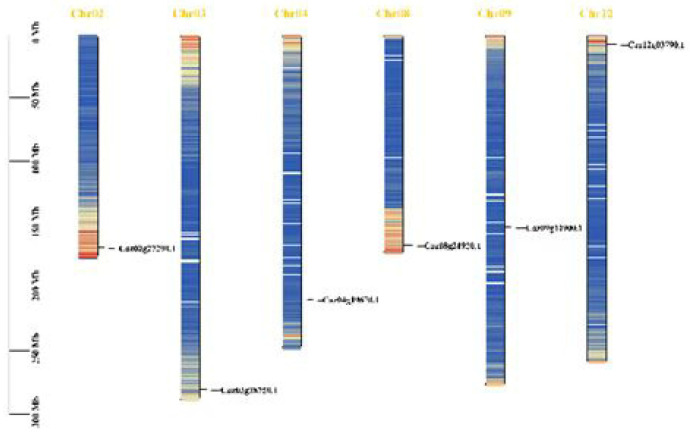
Chromosomal distribution of members of the *ICK*/*KRP* gene family in pepper.

**Figure 3:**
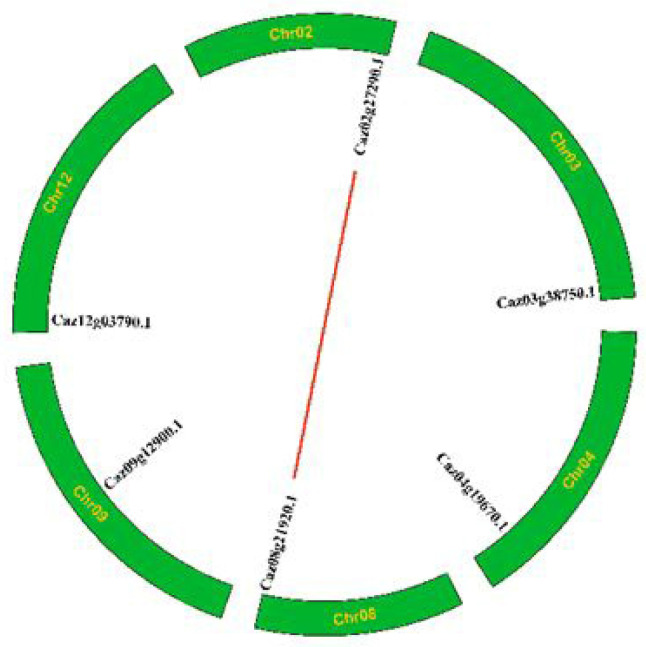
Chromosomal circos plot of pepper *ICK*/*KRP* gene family members.

The chromosomal locations of *SmKRPs* were analyzed. The results showed that the six *KRP* genes were distributed on six different chromosomes, namely chromosomes 2, 3, 4, 8, 9 and 12 (Figure 2). We identified a collinear relationship between *Caz08g21920.1* and *Caz02g27290.1* (Figure 3), which was consistent with the results of the phylogenetic tree, indicating that a segment duplication event had occurred between *Caz08g21920.1* and *Caz02g27290.1*.

### 2.4. Analysis of Gene Structure and Conserved Motifs of ICK/KRP Family Members

**Figure 4:**
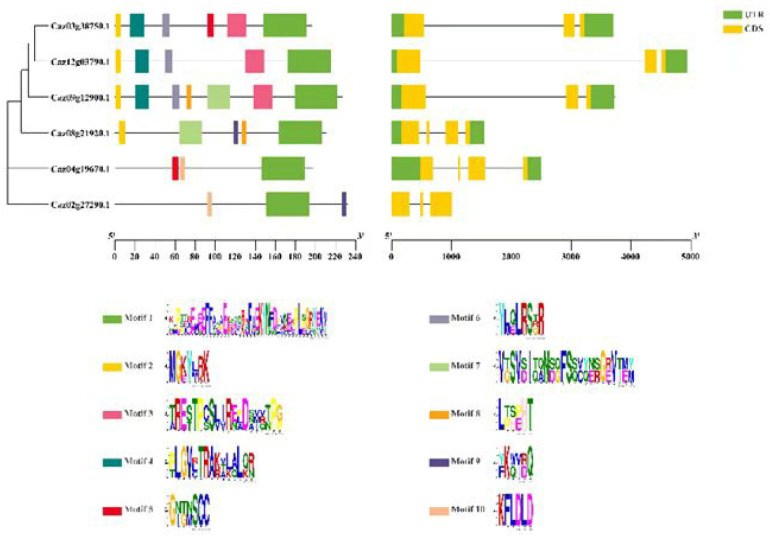
Gene structure and conserved motifs of members of the *ICK*/*KRP* gene family in pepper.

The phylogenetic relationships among Krüppel-like repressor protein (KRP) family members were analyzed. *Caz03g38750.1*, *Caz09g12900.1* and *Caz12g03790.1* exhibited relatively high sequence similarity with each other (Figure 4), and all of them contained two introns and three exons. A total of 10 conserved motifs (motif 1–10) were identified via the Multiple Em for Motif Elicitation (MEME) web server with amino acid sequence search (Table 3). The amino acid sequence lengths of these conserved motifs ranged from 6 to 37 amino acids (aa). Among them, Motif 1 represented the CDI domain, which was present in all six genes as a conserved domain of the KRP family.

### 2.5. Analysis of Cis-Acting Elements in ICK/KRP Family Members

**Figure 5:**
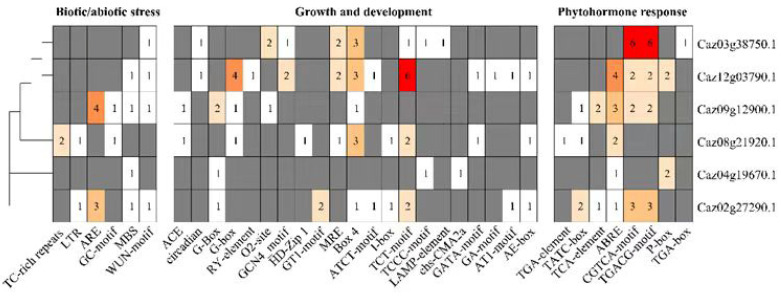
The heatmap of cis-acting element functional enrichment for the pepper *ICK*/*KRP* gene family. The color gradient from white to dark red represents the increasing enrichment level of cis-elements; each column corresponds to a specific cis-regulatory element, and each row represents an individual pepper ICK/KRP gene.

Cis-acting elements are one of the key factors regulating gene expression, thereby affecting the development and stress responses of organisms. The cis-acting elements in the promoters of the *ICK* gene family were analyzed. A total of 81 cis-acting elements associated with biotic/abiotic stress, growth and development, and phytohormone responsiveness were identified in the promoter regions of these six *KRP* genes (Figure 5). Among the biotic/abiotic stress-responsive elements, there were TC-rich repeats involved in defense and stress responses, *MBS* (MYB binding site) involved in drought induction, LTR elements associated with low-temperature responsiveness, and the GC-motif (an enhancer) involved in hypoxia-specific induction. Each of these genes contained no fewer than one stress-responsive element, indicating that the expression of these genes is affected by environmental factors. Growth and development-related elements included ACE, G-Box and MRE elements involved in light responsiveness, HD-Zip 1 elements responsible for regulating palisade mesophyll cell differentiation, and the RY element associated with seed-specific regulation. Elements related to growth and development accounted for 54.5% of the total cis-acting elements, suggesting that these genes play important roles in developmental processes of pepper, such as cell differentiation and seed development. Phytohormone-responsive elements consisted of the TATC-box involved in gibberellin responsiveness, the TCA element associated with salicylic acid responsiveness, ABRE, involved in abscisic acid responsiveness, the CGTCA-motif and TGACG-motif as cis-regulatory elements for MeJA responsiveness, P-box as a gibberellin-responsive element, and TGA-box as an auxin-responsive element. These results indicated that phytohormones exert a considerable regulatory effect on the *ICK* gene family. In particular, the *Caz03g38750.1* gene contained 12 MeJA-responsive elements, implying that jasmonic acid may strongly regulate the expression of this gene.

### 2.6. Analysis of Expression Patterns of the Pepper ICK/KRP Family in Different Tissues and Under Heat Stress Treatment

**Figure 6:**
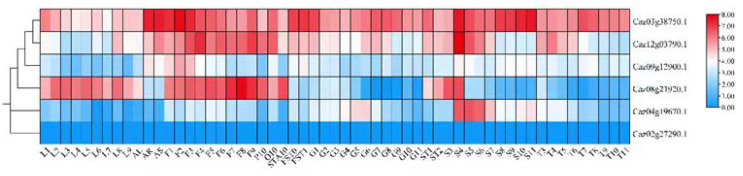
Heatmap of expression levels of the pepper *ICK*/*KRP* gene family in different tissues. L: leaf; F: flower; G: fruit; ST: seed and placenta; S: seed; T: placenta; P10: petal; O10: ovary; STA10: stamen.

To explore the expression profiles of *ICK*/*KRP* genes during pepper development, pepper transcriptome data were downloaded from the Pepper Hub database, and the transcriptome data of leaves, flowers, and fruits at different developmental stages were extracted for the construction of clustered heatmaps via TBtools v1.126 software. The results revealed that the expression levels of pepper *ICK*/*KRP* genes exhibited significant differences in different tissues and at different developmental stages (Figure 6). *Caz03g38750.1* was expressed in all tested tissues, while *Caz02g27290.1* showed low expression levels across all tissues. *Caz08g21920.1* was expressed in leaves and flowers, as well as seeds at the third and fourth developmental stages.

**Figure 7:**
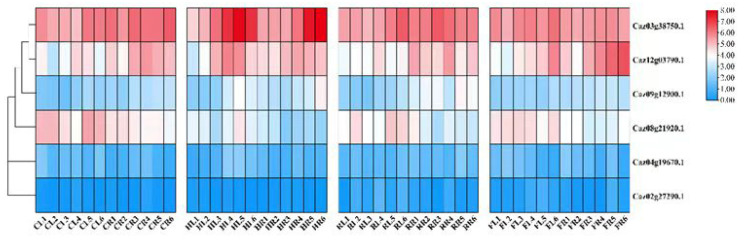
Heatmap of *ICK*/*KRP* family gene expression levels in leaves and roots under different stresses. CL: control leaves; CR: control roots; HL: leaves under heat stress; HR: roots under heat stress; RL: leaves treated with ROS; FL: leaves under cold stress; RR: roots treated with ROS; FR: roots under cold stress.

The expression analysis of pepper *ICK*/*KRP* genes under heat stress (Figure 7) showed that the expression levels of *Caz04g19670.1* and *Caz02g27290.1* were extremely low or completely absent under heat stress. The expression of *Caz03g38750.1* was upregulated in the 4th, 5th and 6th stages after heat treatment, and its expression was also upregulated in roots at the 5th and 6th stages. The expression of *Caz08g21920.1* was downregulated in both leaves and roots after heat treatment. *Caz12g03790.1* was upregulated in roots under cold stress treatment, and *Caz08g21920.1* was downregulated under ROS treatment. These results indicate that different *ICK*/*KRP* genes exhibited distinct responses to heat stress and adopted different expression patterns to adapt to heat stress responses.

### 2.7. Analysis of the Relative Expression Levels of Pepper ICK/KRP Genes

**Figure 8:**
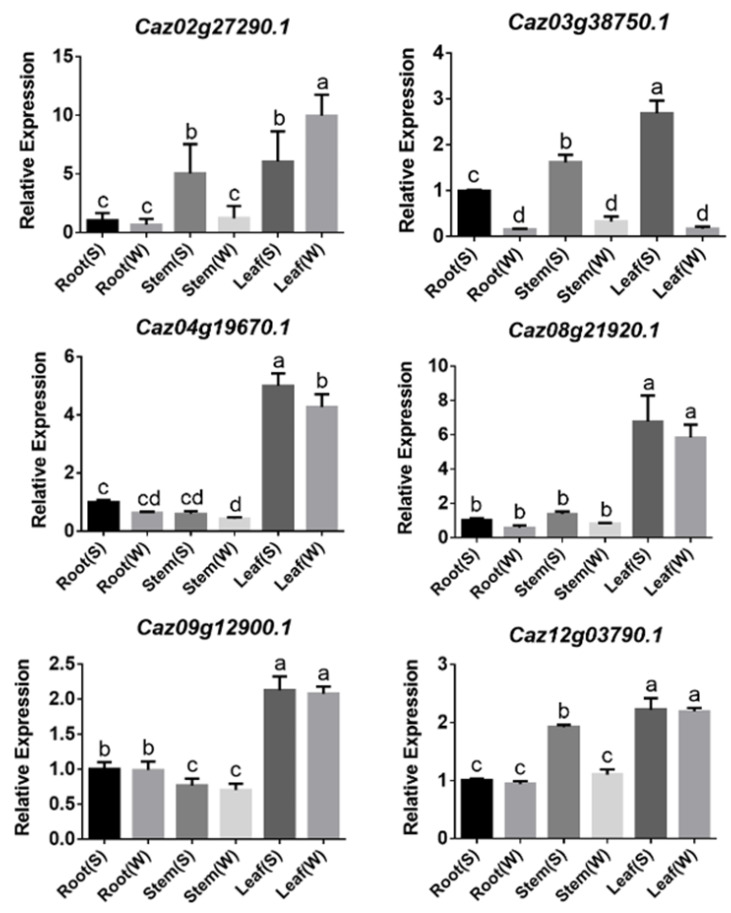
The relative expression level of *ICK*/*KRP* genes in pepper tissues and organs. RT-qPCR analysis was performed to examine the relative expression levels of six *ICK*/*KRP* family genes in the roots, stems, and leaves of the long-stigma pepper line SJ10 (S) and the short-stigma pepper line W1F2 (W). Different lowercase letters above the bars indicate significant differences between groups.

RT-qPCR expression analysis was performed on six *ICK* family member genes in the roots, stems, and leaves of long-stigma pepper SJ10 (S) and short-stigma pepper W1F2 (W) (Figure 8). The results showed that all genes exhibited significant tissue-specific expression patterns, with leaves serving as the primary expression tissue and their relative expression levels markedly higher than those in roots and stems. The expression levels of all genes were relatively low in root tissues, and most genes showed no significant differences between the two varieties. For *Caz02g27290.1*, the highest expression was detected in leaves, followed by stems, and the lowest in roots. In terms of varietal differences, the expression level in the stems of SJ10 was significantly higher than that of W1F2, while in leaves, the expression in W1F2 was remarkably higher than in SJ10, with no significant difference observed in roots between the two varieties.

The expression of *Caz03g38750.1* in all tested tissues of SJ10 was significantly higher than that in W1F2, with the most prominent difference in leaves; this gene maintained an extremely low expression level in the roots, stems and leaves of W1F2. Leaves were the dominant expression tissue for *Caz04g19670.1*, with low expression levels in roots and stems and no significant varietal differences. In leaves, the expression level of SJ10 was significantly higher than that of W1F2. *Caz08g21920.1* and *Caz09g12900.1* shared similar expression profiles, both showing high leaf-specific expression and extremely low expression in roots and stems, with no significant differences between varieties. There was no statistical difference in the expression levels of leaves between the two cultivars. *Caz12g03790.1* showed the highest expression in leaves, followed by stems and roots. Varietal differences were only found in stem tissues, where SJ10 presented significantly higher expression than W1F2, whereas no distinct differences existed in leaves and roots. *Caz03g38750.1* and *Caz04g19670.1* were specifically and highly expressed in the leaves of SJ10, which may be involved in the regulation of the long-stigma trait. *Caz02g27290.1* was highly expressed in the leaves of W1F2 and may be related to the specific physiological regulation of W1F2. In contrast, *Caz08g21920.1* and *Caz09g12900.1* are core functional genes for pepper leaf development, with weak correlation to stigma traits.

**Figure 9:**
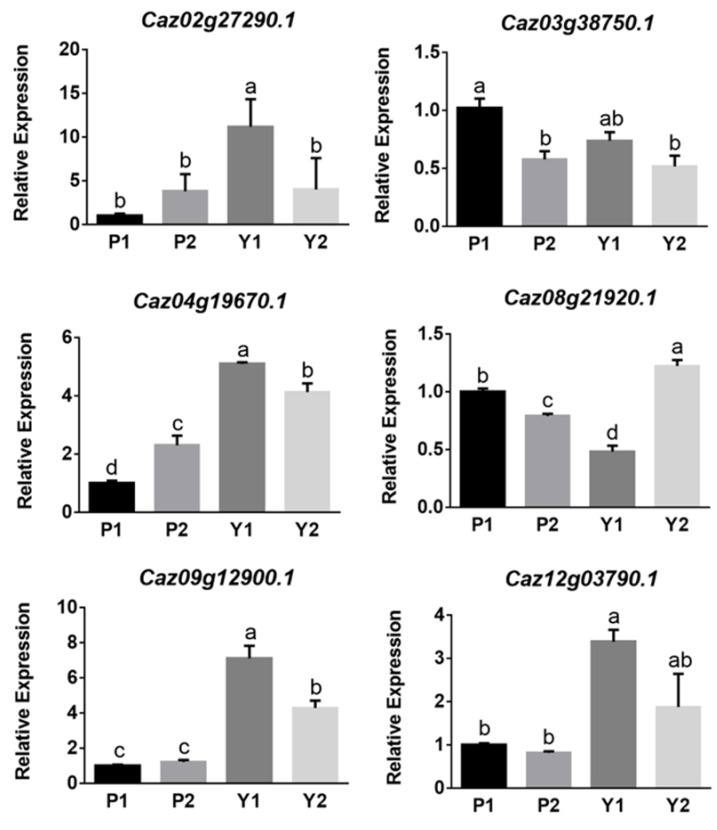
Relative expression levels of *ICK*/*KRP* genes in pepper. P1: Long-stigma pepper, 1 day before flowering; P2: long-stigma pepper after pollination; Y1: short-stigma pepper, 1 day before flowering; Y2: short-stigma pepper after pollination. Different lowercase letters (a, b, c, d) above bars indicate statistically significant differences (*p* < 0.05).

The *ICK* protein can affect cell cycle progression by inhibiting the activity of the CDK–cyclin complex, thereby indirectly influencing cell elongation. To investigate whether *ICK* affects the length of the pollen tube, stigma RNA was separately extracted from two pepper varieties at two developmental stages, including long-stigma peppers at one day before flowering (P1) and after pollination (P2), as well as short-stigma peppers at one day before flowering (Y1) and after pollination (Y2). RT-qPCR validation and expression analysis of *ICK* gene family members were performed on pepper stigmas before and after pollination (Figure 9). The results showed that all *ICK* genes were expressed in pepper stigmas, and the same gene exhibited significant expression differences in different developmental stages and different pepper materials. At one day before flowering, the expression levels of *Caz02g27290.1*, *Caz04g19670.1* and *Caz09g12900.1* in short-stigma materials were higher than those in long-stigma materials, and their expression levels remained higher in short-stigma materials across different stages. After pollination, the expression levels of *Caz02g27290.1*, *Caz04g19670.1*, *Caz09g12900.1*, *Caz08g21920.1* and *Caz12g03790.1* in short-stigma materials were all higher than those in long-stigma materials. In contrast, the expression level of *Caz03g38750.1* in long-stigma materials was higher than that in short-stigma materials both before and after pollination, while the expression pattern of *Caz12g03790.1* showed the opposite trend. These findings suggest that the above genes may jointly regulate the development of stigma length in pepper.

### 2.8. Subcellular Localization Analysis of ICK/KRP Genes in Pepper

**Figure 10:**
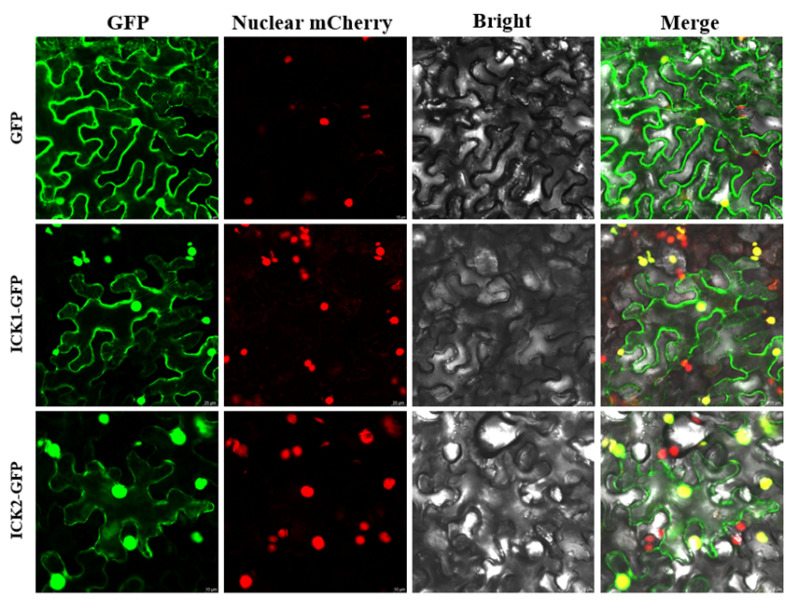
Subcellular localization of *ICK1 (Caz03g38750.1)* and *ICK2 (Caz12g03790.1)* in *N. benthamiana* leaves. The nucleus was indicated by mCherry fused with *ICK1* and *ICK2*. Scale bar = 25 μm. Green fluorescence represents the signal of ICK-GFP fusion protein; red fluorescence indicates mCherry nuclear marker; the merged channel shows the co-localization of two fluorescent signals. The original high-resolution SEM image is provided in the Supplementary Materials.

Based on the results of RT-qPCR, candidate genes were screened for subcellular localization (Figure 10). Transient transformation was performed via Agrobacterium-mediated tobacco infiltration, and the localization of fluorescent proteins was observed under a confocal laser scanning microscope. The results demonstrated that the expression products were localized in the nucleus and cell membrane.

### 2.9. Phenotypic Changes in Pepper Stigma Mediated by VIGS-Induced Silencing of ICK1

**Figure 11:**
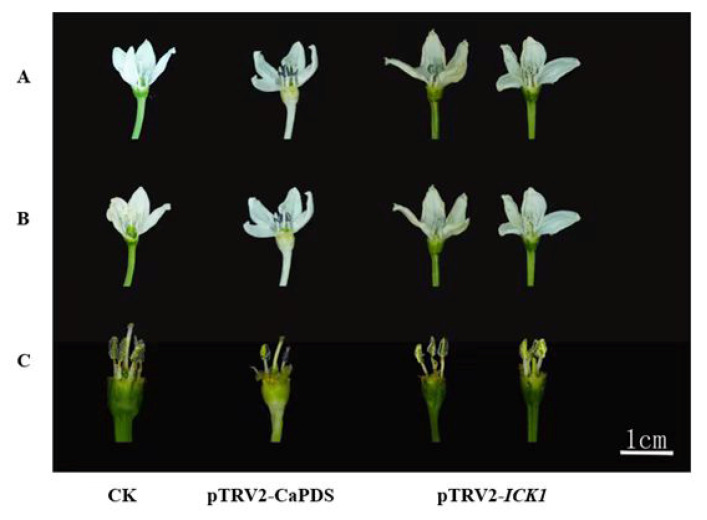
Phenotypes of pepper stigma after silencing *ICK1 (Caz03g38750.1)* genes. (**A**): Front view of intact flowers; (**B**): partial stamens removed; (**C**): petals removed, showing the morphology of stigmas and stamens. CK: Untreated wild-type control; pTRV2-CaPDS: VIGS positive control; pTRV2-*ICK1*: *ICK1*-silenced pepper plants. Scale bar: 1 cm.

To explore the effects of *ICK1* silencing on stigma development in pepper, we performed virus-induced gene silencing (VIGS) of *ICK1* in pepper cultivar ‘SJ10’. Untreated plants (CK) and pTRV2-CaPDS-silenced plants were used as controls, and we conducted systematic morphological observation and quantitative measurement of floral organs and stigma length (Figure 11). The results showed that compared with CK and pTRV2-CaPDS control groups, no obvious changes were observed in the petal morphology of *ICK1*-silenced plants (pTRV2-*ICK1*), whereas pistil development was markedly impaired.

**Figure 12:**
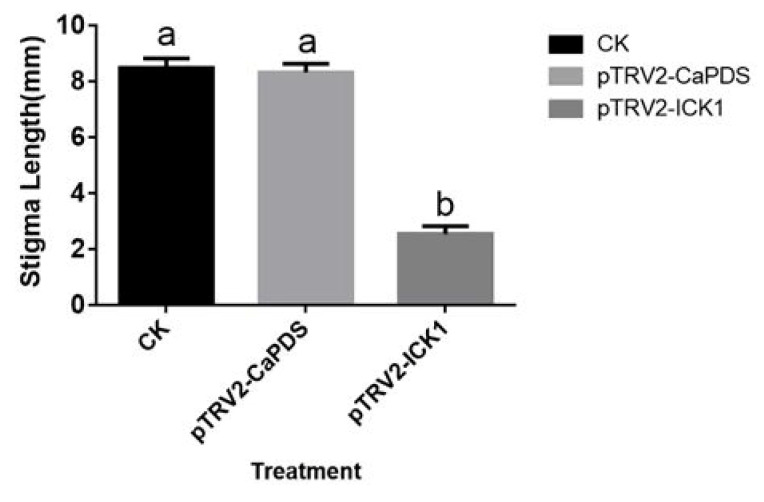
Quantitative analysis of stigma length in pepper under different treatments. CK: wild-type control; pTRV2-CaPDS, positive control; pTRV2-*ICK1*, *ICK1*-silenced plants. Bars represent the mean values, and error bars indicate standard deviation (SD). Different lowercase letters indicate significant differences among groups at *p* < 0.05. The quantitative analysis of stigma length is provided in Supplementary Figure S1.

Quantitative analysis revealed that the stigma length was (8.47 ± 0.35) mm in the CK group and (8.32 ± 0.31) mm in the pTRV2-CaPDS positive control group, with no significant difference between the two groups (*p* > 0.05). By contrast, the stigma length of pTRV2-*ICK1* plants was only (2.54 ± 0.28) mm (Figure 12), which was extremely significantly lower than that of the two control groups (*p* < 0.001). The observation of pistil and stamen structures after petal removal (Figure 11C) indicated that stigmas of CK and pTRV2-CaPDS controls were obviously higher than anthers, showing a typical long-stigma phenotype. However, stigmas of pTRV2-*ICK1* plants were dramatically shortened, with stigma positions close to or lower than anthers, presenting a typical short-stigma phenotype.

Collectively, these results demonstrate that silencing of *ICK1* specifically represses stigma elongation and alters the relative position between stigmas and anthers in pepper.

## 3. Discussion

Cell differentiation and expansion occur throughout the whole process of plant growth and development, regulating processes such as flowering and fruiting, seed development and reproduction, and playing critical roles in responding to environmental changes including heat and cold stress [10]. As inhibitors of CDK protein activity, *ICK*/*KRP*s exert an essential regulatory role in modulating the cell cycle [2]. To date, *ICK*/*KRP* genes have been identified in many plant species, such as *arabidopsis*, *tomato* and *soybean*. A total of seven *ICK*/*KRP* genes have been identified in *arabidopsis*, and functional studies of this gene family have been conducted using RNA interference (RNAi) assays and T-DNA mutants. The results have revealed that there are no significant phenotypic differences between lower-order mutants and wild-type plants, whereas higher-order mutants exhibited altered leaf width and petal and seed size, which have been characterized in this model plant [11]. In crops, *rice* KRP mutants displayed phenotypic traits including reduced seed size and dwarf plants, suggesting that *ICK*/*KRP*s possess a certain degree of functional conservation in the regulation of plant development [12]. During plant growth under stress conditions, both biotic and abiotic stresses can inhibit cell cycle progression and exert adverse effects on plant growth. Since plants are sessile organisms, they have evolved a mechanism to adjust the cell cycle process in response to environmental signals. Studies on tomato have demonstrated that low-temperature stress significantly upregulated the expression level of tomato *SlKRP2*, and drought stress markedly induced the expression of tomato *SlKRP3*; the silencing of *SlKRP5* increased the sensitivity of tomato to drought stress [7]. In soybean, polyethylene glycol (PEG) stress upregulated the expression levels of GmKRP1a, GmKRP2a and GmKRP4, and the expression levels of GmKRP1a, GmKRP2a, GmKRP2b, GmKRP4 and GmKRP5 were elevated under salt stress treatment [8]. Five *KRP* genes have been identified in eggplant, and the expression of SmKRP3 was significantly increased under salt stress. Subcellular localization assays revealed that all eggplant KRPs were localized in the nucleus. The silencing of SmKRP3 enhanced the sensitivity of eggplant to salt stress, accompanied by a significant reduction in the expression of salt stress marker genes SmGSTU10, SmNCED1, SmDHN1 and SmDHNX1 [9].

A total of six *ICK*/*KRP* genes were identified in the whole genome of pepper, representing a relatively small gene number. The number of *ICK*/*KRP* gene family members varies slightly among different plant species, and these genes can essentially be divided into three categories, which is consistent with previous studies [13]. From the perspective of monocot and dicot classification, clade A genes are unique to *rice* (a monocot plant), implying that this category of genes may have played important roles in the evolutionary process. The six *ICK*/*KRP* family genes were distributed on different chromosomes. Collinearity analysis revealed that *Caz02g27290.1* and *Caz08g21920.1* are homologous duplicated genes. However, expression analyses across different tissues and stress conditions demonstrated that these two genes exert divergent functions in plant development and stress responses, showing differentiated expression patterns. Promoter cis-acting element analysis showed that *Caz02g27290.1* contained 17 cis-acting elements while *Caz08g21920.1* harbored 14 cis-acting elements, with the main difference lying in the MeJA-responsive elements (CGTCA-motif and TGACG-motif). We analyzed the expression patterns of six genes from the pepper *ICK*/*KRP* family in different tissues of the long-stigma line SJ10 and the short-stigma line W1F2. The results showed that these genes exhibited clear tissue-specific expression and significant differences between the two genotypes. Most *ICK* genes were expressed at significantly higher levels in leaves compared to roots and stems, and their expression levels in the leaves of the long-stigma line SJ10 were significantly higher than those in the short-stigma line W1F2. This is highly consistent with the functional characteristics of *ICK* genes as negative regulators of the cell cycle. Based on transcriptome data and quantitative expression validation, *Caz03g38750.1* exhibited a higher expression level in long-stigma materials before and after pollination, whereas *Caz12g03790.1* showed the opposite expression pattern, with higher expression in short-stigma materials. These results suggest that the two genes may cooperatively regulate the development of stigma length in pepper.

Stigma length in pepper affects the rates of self-pollination and cross-pollination in plants, as well as the reproductive population structure of the pepper. Pepper plants with short stigmas are prone to self-pollination, whereas those with long stigmas can easily capture exogenous pollen and thus enhance the cross-pollination rate [14]. At present, molecular regulatory studies on stigma exsertion in solanaceous crops have mostly focused on tomato and *rice*, while relevant research in pepper remains relatively scarce. Wu et al. [15] successfully cloned the HD-ZIP IV transcription factor, which can directly regulate the expression of Style 2.1, a key determinant transcription factor of style length in tomato. Studies have revealed that the HD-ZIP IV transcription factor is highly expressed in the apical region of the style, and it regulates the endoreduplication process of stylar cells through a concentration-dependent dosage effect, thereby mediating the polar elongation and growth of the style. Shang et al. [16] found that the SE3.1 and Style 2.1 genes mediate the morphological evolution of tomato stigmas through a two-step regulatory model via synergistic effects: the mutation of Style 2.1 changes stigmas from exserted to flush, and the mutation of SE3.1 further causes stigma retraction. This regulatory pattern drives the transition of tomato from cross-pollination to self-pollination. Zhu et al. [17] screened GS3, GW8 and GS9 as key candidate genes for stigma exsertion in *rice* using expression analysis and other techniques, and confirmed that the expression trends of these three genes were consistent with the developmental trends of *rice* stigmas. Subsequently, single, double and triple mutants of these three genes were generated using the CRISPR/Cas9 gene editing technology. Experimental results showed that the stigma exsertion rate of the triple mutant plants increased to more than 50%, with no adverse effects on the normal growth and agronomic traits of *rice*. The *rice* TGW2 protein can interact with the cell cycle regulatory protein KRP1, and this interaction negatively regulates grain width and grain weight in *rice*. After introducing the superior allelic variant tgw2 into target *rice* varieties, the grain yield was increased by 12.3% [5]. The results of subcellular localization in this experiment indicated that the encoded proteins of *ICK1* (*Caz03g38750.1*) and ICK2 (*Caz12g03790.1*) were dual-localized in the nucleus and plasma membrane of pepper style cells. As core cell cycle regulators, the proteins execute the function of inhibiting cell proliferation in the nucleus; meanwhile, they participate in the perception and transduction of growth and polarity signals on the plasma membrane. The combination of these two subcellular locations enables the two genes to link signal response and cell cycle control, laying a structural foundation for their dual regulation of intracellular cell cycle signaling and transmembrane developmental signal transduction. We also found that two genes of the *ICK*/*KRP* family exhibited differential expression in materials with long and short styles, which respond to style length variation and function as key upstream regulatory factors mediating stigma developmental differences in pepper. This study silenced the *ICK1* gene in pepper using VIGS technology and found that the stigma length of silenced plants was significantly reduced, indicating that *ICK1* specifically participates in the regulation of stigma elongation in pepper. As core negative regulatory factors of the plant cell cycle, *ICK*/*KRP* family proteins execute precise cascade regulation on cell cycle progression: they specifically bind to and inhibit the kinase activity of CDK–Cyclin complexes, block the G1/S and G2/M phase transition of the cell cycle, restrain excessive cell proliferation, and control the division rate and proliferation amplitude of style cells. Stigma elongation depends on the ordered division and polar expansion of style cells. Normal stigma development requires a dynamic balance between cell division and cell polar expansion. The moderate inhibitory effect of *ICK1* on the cell cycle can terminate redundant cell division in the late stage of style development, avoid disordered cell proliferation, and create favorable spatial and physiological conditions for subsequent longitudinal polar expansion of cells. The high expression of *ICK1* in the style/leaves of the long-stigma line SJ10 and the inhibition of stigma elongation after silencing are highly consistent with the functional characteristics of *ICK* genes in regulating the cell cycle. It is speculated that *ICK1* may maintain cell cycle homeostasis by finely regulating the division rate and expansion process of style cells, thereby ensuring normal stigma elongation. Specifically, the high basal expression of *ICK1* in long-stigma materials can precisely modulate CDK–Cyclin complex activity, maintain orderly and moderate cell division, and promote the transformation of style cells from a proliferation state to an elongation state, thus driving continuous stigma elongation. When the expression of this gene is silenced, the inhibitory effect on CDK–Cyclin complexes is weakened, resulting in abnormal activation of cell cycle progression, disordered proliferation of style cells, and failure of normal polar expansion and longitudinal growth. When the expression of this gene is silenced, the regulation of the cell cycle in style cells is disrupted, leading to insufficient cell division and expansion and ultimately resulting in shortened stigma length. These results provide direct functional evidence for the involvement of the *ICK1* gene in the regulation of pepper stigma development, offering an important candidate gene for analyzing the molecular mechanism of stigma length regulation and improving pollination efficiency in pepper. This study further clarifies the complete regulatory pathway of *ICK1*-mediated cell cycle balancing and stigma elongation, revealing a new molecular mechanism: *ICK1* controls pepper stigma morphological development via targeting the CDK–Cyclin cell cycle core complex, which enriches the molecular regulatory network of plant reproductive organ development.

Stigma exsertion is a quantitative trait, and the genes controlling stigma exsertion vary among different pepper materials. This trait is also affected by external environmental factors. The stigma exsertion rate is an important index for hybrid seed production, and a high stigma exsertion rate is a key trait to improve the outcrossing seed setting rate. Breeding male sterile lines with stigma exsertion can greatly simplify the process of hybrid seed production, and increasing the proportion of long-stigma flowers can reduce flower and fruit abscission caused by short-stigma flowers. Regulating the stigma exsertion trait through cultivation measures can significantly improve pepper yield and quality, enhance the efficiency of insect pollination, and accelerate the breeding process of new pepper varieties with superior quality, high yield and strong adaptability.

## 4. Materials and Methods

### 4.1. Identification of the ICK/KRP Gene Family in Pepper

The genome of Zhangshugang pepper (https://ted.bti.cornell.edu/cgi-bin/pepper/search (accessed on 10 January 2026)) was used as the reference genome, from which the protein sequence file and GFF annotation file were downloaded. Based on the conserved-domain profile (PF02234, associated with FAD-binding) corresponding to the conserved domain of the *ICK*/*KRP* family, the corresponding Hidden Markov Model (HMM) file was downloaded from the Pfam database (Pfam Consortium, European Bioinformatics Institute (EBI), Hinxton, UK; http://pfam-legacy.xfam.org, accessed on 10 January 2026). The HMMER v3.3.2 software (Eddy Lab, Harvard University, Cambridge, MA, USA) was employed in the local PowerShell environment to perform homologous sequence screening against the protein sequences of the pepper genome, thereby identifying the members of the chili pepper *ICK*/*KRP* gene family.

### 4.2. Analysis of Physicochemical Properties of the ICK/KRP Gene Family in Pepper

The physical and biochemical characteristics of the target proteins were analyzed using the ExPASy ProtParam (web 2024 version) online tool (Swiss Institute of Bioinformatics, Lausanne, Switzerland; https://web.expasy.org/protparam/, accessed on 10 January 2026) [18]; and the physicochemical properties of the pepper *ICK*/*KRP* gene members, including the number of amino acids, molecular weight, hydrophilicity, isoelectric point, instability index and aliphatic index, were obtained. Subcellular localization prediction was performed using the Plant-mPLoc 2.0 online platform (Center for Bioinformatics, Shanghai Jiao Tong University, Shanghai, China; http://www.csbio.sjtu.edu.cn/bioinf/plant-multi/, accessed on 10 January 2026) [19].

### 4.3. Procedures for Phylogenetic Tree Construction of ICK/KRP Proteins from Pepper, Tomato, Rice, Maize and Arabidopsis

Multiple sequence alignment of the *ICK* proteins from pepper, tomato, *rice*, *maize*, and *arabidopsis* was performed using the ClustalW 2.1 software (Conway Institute of Biomolecular and Biomedical Research, University College Dublin, Dublin, Ireland). A phylogenetic tree was constructed with the MEGA 7.0 software by adopting the neighbor-joining method, with the number of bootstrap replicates set to 1000, and other parameters kept as default values.

### 4.4. Chromosomal Localization of the ICK/KRP Gene Family in Pepper

The PhenoGram v2.0 tool (Center for Computational Biology, Johns Hopkins University, Baltimore, MD, USA) was employed for chromosomal localization analysis and mapping of the *KRP* gene family [20]. Meanwhile, the gene localization visualization module of the GTF/GFF function in TBtools software was used to analyze the chromosomal distribution characteristics of *ICK*/*KRP* genes [21]. Additionally, the integrated Multiple Collinearity Scan toolkit (MCScanX) was utilized to analyze and visualize the collinearity relationships of *ICK*/*KRP* genes [22].

### 4.5. Gene Structure and Conserved Motif Analysis of the ICK/KRP Gene Family in Pepper

Genomic sequences, CDS sequences, protein sequences, and exon/intron structural information of all candidate *KRP* genes were extracted from genomic data. Subsequently, the TBtools software was used to draw and visualize the schematic diagrams of gene structures [23]. The Simple MEME Wrapper module in the TBtools-II software package (Version 2.083) was adopted for motif type analysis [23], and the Gene Structure View module was utilized to achieve integrated visualization of motif types and gene structures.

### 4.6. Cis-Acting Element Analysis of the ICK/KRP Gene Family in Pepper

The Basic module for gene structure visualization in TBtools software was used to verify the structural characteristics of *ICK*/*KRP* genes again. Meanwhile, the PlantCARE database (http://bioinformatics.psb.ugent.be/webtools/plantcare/html/) was employed to search and identify the cis-acting elements in the promoter regions of ICK/KRP genes [24].

### 4.7. Expression Pattern Analysis of the ICK/KRP Gene Family in Pepper

The raw transcriptome data of different pepper tissues were retrieved from the NCBI database (PRJNA193077), while the transcriptome data of pepper under hormone and abiotic stress treatments were obtained from the PepperHub specialized bioinformatics platform (College of Horticulture & Forestry Sciences, Huazhong Agricultural University, Wuhan, China; https://www.pepperhub.in/?srsltid=AfmBOoojkc9E7JVH8Iy7yIfmKNxqUYZ5bO5SXPQaZRhIGakd9t4ULNMm, accessed on 10 January 2026). After acquiring the above transcriptome data, the HeatMap module in the TBtools-II v2.083 software package (Chen Lab, South China Agricultural University, Guangzhou, China) [23] was utilized to perform standardization and cluster analysis on the expression levels of *ICK*/*KRP* family genes in different tissues, under hormone treatments and abiotic stress conditions, and to generate visualized heatmaps. This was conducted to systematically clarify the tissue-specific expression patterns and stress-responsive expression characteristics of this gene family.

### 4.8. Plant Materials Selection and Quantitative Real-Time PCR

Floral organs of pepper materials with long and short stigmas were sampled before and after pollination, following the sampling protocol described by Liu et al. [25]. Meanwhile, stigma tissues were collected 1 day before flowering and 1 day after pollination (see the attached figure for specific sampling sites). All samples were quickly ground in liquid nitrogen, and total RNA was extracted using the Vazyme Polysaccharide and Polyphenol-rich Plant Total RNA Extraction Kit (Cat. No.: RC401, Vazyme Biotech Co., Ltd., Nanjing, China). cDNA was synthesized via reverse transcription with the Vazyme One Step RT-qPCR Kit (Cat. No.: Q221, Vazyme Biotech Co., Ltd., Nanjing, China). Primers for the pepper *ICK* gene family were designed using the Primer-Blast tool in the NCBI database [26] (Table 4), with the primer length set at 20–23 bp, Tm value ranging from 57 to 61 °C, and amplified fragment length at approximately 82–120 bp. The pepper Actin gene was used as the reference gene. The reaction system and procedures were configured according to the instructions of the AceQ Universal SYBR qPCR Master Mix Kit (Cat. No.: Q511, Vazyme Biotech Co., Ltd., Nanjing, China) for quantitative real-time PCR experiments, with three biological replicates set for each sample. To ensure data reproducibility, each biological replicate was further equipped with three independent technical replicates. The 2^−ΔΔCT^ method [27] was adopted to calculate the relative expression levels of each *ICK* family member. All data were presented as mean ± standard deviation (SD). Independent sample *t*-tests were performed for statistical significance analysis, and a *p*-value less than 0.05 was considered statistically significant, while *p* < 0.01 indicated an extremely significant difference, so as to systematically analyze the expression patterns of this gene family in different floral development stages and stigma tissues of pepper.

### 4.9. Subcellular Localization

Based on the results of real-time fluorescence quantitative PCR, candidate genes were selected for subcellular localization analysis. The fusion expression vector of the target gene and GFP was constructed, and Agrobacterium-mediated transient transformation was performed on tobacco leaves. The fluorescence signals were observed under a confocal laser scanning microscope to determine the protein localization.

### 4.10. VIGS Analysis

Using tobacco rattle virus (TRV)-mediated virus-induced gene silencing (VIGS), specific fragments of *ICK1* and the reporter gene CaPDS from pepper (*Capsicum annuum*) were cloned into the pTRV2 vector. The resulting constructs, together with pTRV1, were transformed into Agrobacterium tumefaciens strain GV3101. Agrobacterium cultures carrying pTRV1 and each pTRV2-derived vector were mixed at a 1:1 ratio (OD_600_ = 0.8–1.0) and infiltrated into the cotyledons of 2–4 leaf-stage pepper seedlings using a needleless syringe. Wild-type plants (CK) served as the blank control, and plants inoculated with pTRV2-CaPDS were used as the positive control. After 3–4 weeks of cultivation post-inoculation, flower organ phenotypes were observed. The VIGS experiment was performed with three independent biological replicates. Each treatment group and control group contained no fewer than 15 uniformly growing pepper individuals for phenotypic observation and stigma length measurement. All phenotypic data were expressed as mean ± SD. An independent samples *t*-test was used to compare the phenotypic differences between *ICK1*-silenced plants and control plants, with *p* < 0.05 defined as a significant difference.

## Figures and Tables

**Table 1:** Physical and chemical properties of the six *ICK*/*KRP* genes in pepper.

Accession Number	Amino Acids (aa)	Molecular Weight	Grand Average of Hydropathicity	Theoretical pI	Instability Index	Aliphatic Index	Subcellular Localization
*Caz02g27290.1*	232	26,256.5	−0.608	5.84	55.47	69.4	Nucleus.
*Caz03g38750.1*	196	21,761.67	−0.826	9.05	40.02	63.72	Nucleus.
*Caz04g19670.1*	197	22,373.77	−0.917	4.32	72.06	54.92	Nucleus.
*Caz08g21920.1*	211	23,603.02	−0.87	4.77	47.6	53.65	Nucleus.
*Caz09g12900.1*	227	24,984.67	−0.605	6.53	59.17	65.73	Nucleus.
*Caz12g03790.1*	220	24,565.48	−1.03	8.67	55.89	52.73	Nucleus.

**Table 2:** Chromosomal localization of five core members of the *ICK*/*KRP* gene family in *arabidopsis*.

Gene Name	Alias	TAIR Gene ID	Chromosomal Localization	Physical Location (bp)	Strand Orientation	References
*AtKRP1*	*ICK1*	AT2G23430	2	9,234,521–9,236,285	+	Wang et al., 1998 (The Plant Journal)
*AtKRP2*	*ICK2*	AT3G50640	3	18,542,103–18,544,012	−	De Veylder et al., 2001 (Plant Cell)
*AtKRP3*	*ICK3*	AT5G48820	5	21,301,457–21,303,201	+	Jakoby et al., 2006 (BMC Genomics)
*AtKRP4*	*ICK4*	AT2G30960	2	11,987,654–11,989,328	−	Zhou et al., 2018 (PLOS Genetics)
*AtKRP5*	*ICK5*	AT1G49620	1	19,753,841–19,755,603	+	Barrôco et al., 2006 (Plant Physiology)

**Table 3:** Protein motifs of members of the *ICK*/*KRP* gene family in pepper.

Motif ID	Motif Sequence	Length (aa)
Motif 1	KIPTTREIEEFFATAEKQQQRRFIEKYNFDPVNEKPL	37
Motif 2	MGKYLRK	7
Motif 3	TRESTPCSLIREPDSVVTPG	20
Motif 4	PLGVRTRAKVLALQR	15
Motif 5	GNTNSCC	7
Motif 6	YLQLRSTR	8
Motif 7	VTSVSITQNSQFSSVYNSGRVTMY	24
Motif 8	LTSPHT	6
Motif 9	YKWVRQ	6
Motif 10	KFLDLD	6

**Table 4:** Oligonucleotide sequences for primers used in qRT-PCR.

Gene Name	Forward Primer	Reverse Primer
Actin	ATTGGGATGGAAGCTGCGGG	CCAGGGAACATGGTGGAGCC
*Caz02g27290.1*	TGAATTTGCCTGTGGTAACAGAT	TTGTCGCACCCACTTGTAGC
*Caz03g38750.1*	GGAGAGCACACCTTGCAGTT	CTTGCCTTGAGTTCCCCTCA
*Caz04g19670.1*	CCAGCGACAACGCAAAAGAG	AATCCCGGCTGGTTCATTCG
*Caz08g21920.1*	AGTTTCCACCAGAAGCGGAG	GTCCCTCTAATGGCGCATCC
*Caz09g12900.1*	ACTCAAGCGCAACAGCTCAA	TCTCCATAAAAAGGCGCTGC
*Caz12g03790.1*	GGTCCAGAAGAGAAGCAGCA	CCCATTCATAGCGTCCAGGG

## Data Availability

The datasets generated and analyzed during the current study are available from the corresponding author upon reasonable request.

## Supplementary Materials

The following supporting information can be downloaded at: https://www.mdpi.com/article/10.3390/genes17070733/s1; Figure S1: Quantitative analysis of stigma length in pepper under different treatments. CK: wild-type control; pTRV2-CaPDS, positive control; pTRV2-*ICK1*, *ICK1*-silenced plants. Bars represent the mean values, and error bars indicate standard deviation (SD). Different lowercase letters indicate significant differences among groups at *p* < 0.05.
