# Supplementary material for: Identification and Expression Analysis of the Cyclin-Dependent Kinase Inhibitor ICK/KRP Gene Family in Pepper
Source: Genes (Basel). 2026 Jun 25;17(7):733. doi: 10.3390/genes17070733 (PMC13408456; doi:10.3390/genes17070733)
Supplement: Supplementary file 1 [file genes-17-00733-s001.zip › genes-4316105-Supplementary_ Figure.pdf]

Supplementary Figure S1

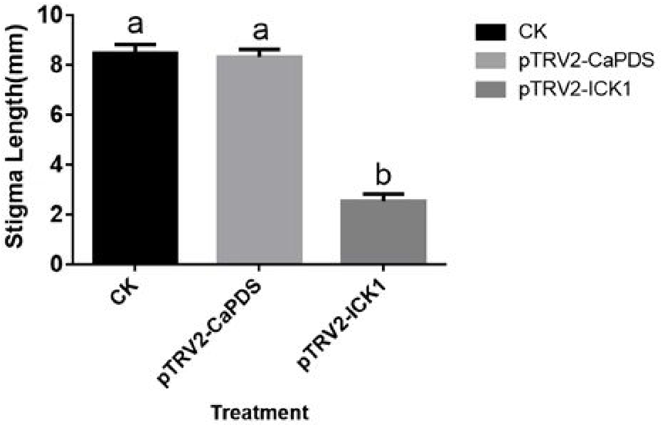

Figure S1. Quantitative analysis of stigma length in pepper under different treatments. CK, wild-type control; pTRV2-CaPDS, positive control; pTRV2-ICK1, *ICK1*-silenced plants. Bars represent the mean values, and error bars indicate standard deviation (SD). Different lowercase letters indicate significant differences among groups at $P<0.05$.
